# The Price of a Whipple: Predicting Hospital Charges Using Preoperative Patient Characteristics

**DOI:** 10.1002/wjs.70286

**Published:** 2026-02-25

**Authors:** Sri Snehita Reddy Bonthu, Sourodip Mukharjee, Joshua Kong, Juan Malo, Houssam Osman, D. Rohan Jeyarajah

**Affiliations:** ^1^ Department of Surgery Anne Burnett School of Medicine at Texas Christian University Fort Worth Texas USA; ^2^ Department of Surgery Methodist Richardson Medical Center Richardson Texas USA

**Keywords:** financial burden, pancreaticoduodenectomy, risk stratification, Whipple surgery

## Abstract

**Background:**

Whipple's pancreaticoduodenectomy (PD) is a highly complex hepato‐pancreato‐biliary (HPB) procedure associated with substantial morbidity and cost. Although postoperative complications are known to increase healthcare expenditures, few studies have explored the association between preoperative factors and healthcare costs. This study evaluated the predictive value of baseline patient characteristics and preoperative laboratory tests in estimating index admission charges for Whipple's PD.

**Methods:**

A retrospective review of 375 patients who underwent open PD (2018–2023) at a high‐volume, non‐university tertiary care center was conducted. Preoperative demographics, comorbidities, laboratory values, and index admission hospital charges were analyzed. Univariate logistic regression identified significant predictors of charges, and the Kruskal‐Wallis test was used to assess the relationship between cumulative preoperative indicators and charges.

**Results:**

Six preoperative laboratory values were significantly associated with increased charges: white blood cell count, hemoglobin, serum albumin, platelet count, prothrombin time, and hemoglobin A1c. A significant association was found between the cumulative preoperative indicators and hospital charges (*p* = 0.001). Patients with three or more unfavorable preoperative indicators incurred significantly higher charges compared to those with fewer than three indicators (*p* < 0.001).

**Conclusion:**

Preoperative laboratory tests, many of which are modifiable, are significant predictors of hospital charges in patients undergoing PD. A higher cumulative burden of adverse preoperative indicators is associated with higher financial charges. These findings support the use of preoperative risk stratification and optimization to enhance value‐based care, guide resource allocation, and mitigate financial toxicity in high‐risk surgical patients.

## Graphical abstract

Preoperative lab abnormalities are significant predictors of hospital charges following Whipple's pancreaticoduodenectomy. In a retrospective review of 375 cases, patients with ≥ 3 preoperative laboratory‐based predictors incurred substantially higher charges, underscoring the value of preoperative optimization in reducing financial burden.

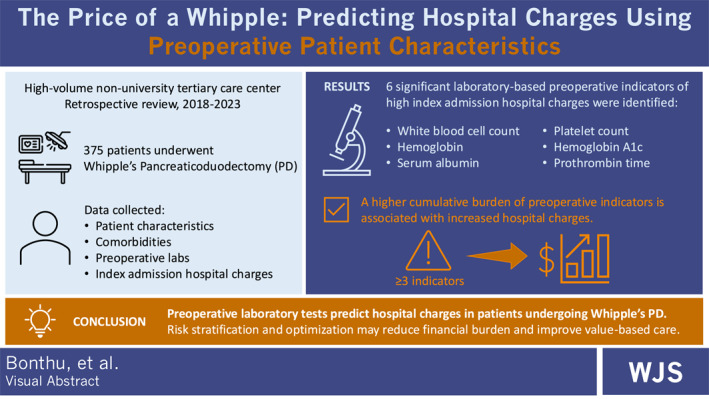

## 1 Introduction

In recent years, healthcare costs have been rising at an alarming rate, with surgical care accounting for nearly one‐third of overall healthcare expenditures [1]. These costs include facility and surgeon fees, anesthesia, pre‐operative testing, ward care, medications, postoperative care, intensive care unit admissions, and allied health services. Postoperative complications can dramatically increase these already high costs by prolonging hospital stays, which often necessitate multidisciplinary management and increased resource allocation [2, 3]. Given the substantial financial burden of surgical care, especially in high‐risk procedures, any data that might help predict and mitigate expenditure will be helpful.

Pancreatic surgeries, particularly the Whipple's pancreaticoduodenectomy (PD), are among the most complex and high‐risk operations performed. Primarily indicated for pancreatic cancer, chronic pancreatitis, or other malignant and premalignant conditions of the pancreas and periampullary region, PD procedures are associated with considerable morbidity and mortality despite advances in surgical technique and perioperative care [4, 5]. When complications occur in PD, this not only impacts patient outcomes but also exerts considerable financial strain on healthcare systems. The medical consequences of complications can lead to delays in chemotherapy, which can affect overall survival.

In addition to system‐level expenses, high‐cost procedures such as PD can lead to financial toxicity, described as financial distress or hardship experienced by patients due to medical care [6]. Financial toxicity is associated with treatment non‐adherence, delayed recovery, and diminished quality of life, all of which lead to poor health outcomes [7]. In hepato‐pancreato‐biliary (HPB) surgery, patients undergoing aggressive interventions, such as PD, may face high out‐of‐pocket expenses, loss of income during recovery, and long‐term financial instability. Identifying and quantifying the factors that drive these costs are essential for addressing patient‐centered outcomes.

Lifestyle‐related factors such as smoking and elevated body mass index (BMI) are associated with poor postoperative outcomes in pancreatic surgery [8, 9]. In addition, various patient characteristics, including age, sex, functional status, comorbid conditions, and American Society of Anesthesiologists (ASA) physical status, are also significant predictors of postoperative complications [10, 11]. These factors increase the risk of complications, which necessitate additional diagnostics, therapeutic interventions, and prolonged hospital stays, ultimately resulting in higher healthcare costs.

Despite the growing awareness of these clinical risk factors, there have been limited studies evaluating their impact on healthcare costs in the context of pancreatic surgery. The existing literature focuses on the overall costs of PD and the financial implications of postoperative complications; however, the connection between preoperative variables that contribute to these complications and their economic consequences remains understudied. Addressing this gap is beneficial in the context of evolving healthcare models, such as value‐based care and bundled payment systems, which aim to optimize both clinical outcomes and cost efficiency [12, 13].

This study aimed to investigate the relationship between preoperative variables, commonly associated with postoperative complications, and their impact on hospital charges in patients undergoing PD. By identifying and evaluating individual and cumulative predictors of hospital charges, this study sought to advance understanding of the financial burden associated with PD, provide actionable insights to reduce costs, optimize preoperative risk assessment, and guide value‐based care strategies.

## 2 Methods

### 2.1 Study Design

A retrospective chart review was performed at Methodist Richardson Medical Center, a high‐volume non‐university tertiary care center (NUTCC), under the approval of the institutional review board. All patients who underwent open PD between 2018 and 2023 by three HPB surgeons were identified through the electronic medical record using Current Procedural Terminology (CPT) codes corresponding to the Whipple procedure and underwent a detailed chart review. The requirement for informed consent was waived due to the retrospective nature of this study, as approved by the IRB for this study. This study was conducted in accordance with the principles outlined in the Declaration of Helsinki.

Demographic information, comorbidities, and preoperative laboratory values were the primary focus of data collection. Patient characteristics, including age, sex, body mass index (BMI), preoperative American Society of Anesthesiologists (ASA) physical status classification, and pancreatic duct size and gland texture, were recorded. Pancreatic duct size and gland texture were recorded intra‐operatively. Pre‐existing comorbidities included a history of smoking, diabetes, and hypertension. Preoperative laboratory parameters included hemoglobin A1c, hemoglobin, platelet count, serum albumin, serum creatinine, white blood cell count, and prothrombin time (PT).

Total in‐hospital charges were obtained from the institutional financial department. The reported charges represent the cost of the index hospital encounter before adjustments and cost discounting and account for room charges, surgical charges, supplies, drugs, labor, equipment, and facility costs. Charges for readmission or reoperation were not included in the analysis. Total in‐hospital charges were adjusted for inflation to December 2024 U.S. dollars using the U.S. Department of Labor Consumer Price Index Inflation Calculator [14]. Financial data are presented in Dollar Equivalents (DE), where one DE corresponds to $150,000. This value was calculated from the median adjusted total hospital charges for the cohort.

### 2.2 Statistical Analysis

The effect of the preoperative variables on index admission hospital charges was compared using the Mann‐Whitney *U* test, and results were reported as medians. Continuous variables were dichotomized using Youden's index to maximize sensitivity and specificity. Univariate logistic regression analyses were used to identify preoperative variables significantly associated with total index admission hospital charges. A cumulative risk score was derived from statistically significant preoperative indicators of increased hospital charges for each patient. Kruskal‐Wallis test was performed to evaluate the association between the total hospital charges and the cumulative risk score. Cumulative preoperative indicators were further categorized into low‐risk (< 3 preoperative indicators) and high‐risk (> 3 preoperative indicators) groups for risk stratification, and their association with postoperative outcomes was examined using the Mann‐Whitney *U* test. The significance level was set at *p* < 0.05. Data were analyzed using IBM SPSS Version 29 (Predictive Analytics Software, Armonk, NY, USA).

## 3 Results

### 3.1 Preoperative Demographics and Characteristics

A total of 375 patients underwent an open PD between January 1, 2018, and December 31, 2023. The median age of the patients was 67 years, and 200 (53.5%) were male, while 174 (46.5%) were female. The median BMI was 26.6 kg/m^2^, with 115 patients (30.8%) classified as obese (BMI > 30). A total of 202 patients (54.7%) had a smoking history (current or prior smokers), 127 (34%) had diabetes mellitus, and 208 (56%) had hypertension. The median pancreatic duct size was 3.0 mm, and of the 133 patients whose gland texture was recorded, 58 (43.6%) had soft texture while 75 (56.4%) had firm texture. Patient demographics and preoperative variables are summarized in Table 1.

**TABLE 1:** Demographic breakdown, past medical history, and preoperative labs of patient cohort.

Variables		Statistic *N* (%) or median (IQR)
Age (median, IQR)		67 (59–74)
Sex	Male	200 (53.3%)
	Female	174 (46.4%)
BMI (median, IQR)		26.6 (23.75–31.13)
ASA	2	60 (16.0%)
	3	244 (65.1%)
	4	71 (18.9%)
Smoking history		167 (45.3%)
Diabetes		127 (34.0%)
Hypertension		208 (55.8%)
WBC preop (median, IQR)		5.90 (5.7–8.5)
Hemoglobin pre‐op (median, IQR)		13.1 (10.8–13.5)
Platelets pre‐op (median, IQR)		234 (200–306)
PT pre‐op (median, IQR)		13.1 (12.9–14.2)
Albumin pre‐op (median, IQR)		4.0 (3.4–4.3)
HbA1c pre‐op (median, IQR)		5.30 (5.3–6.68)
Pancreatic duct size		3.0 (1.0–5.0)
Pancreatic texture	Soft	58 (43.6%)
	Firm	75 (56.4%)

Abbreviations: ASA, American Society of Anesthesiologists; BMI, body mass index; INR, international normalized ratio; PT, prothrombin time; WBC, white blood cell.

### 3.2 Preoperative Variables

Univariate logistic regression analyses identified several preoperative laboratory values as statistically significant predictors of the total hospital charges for index admission (Table 2). Preoperative white blood cell count (*p* = 0.041), hemoglobin level (*p* < 0.001), serum albumin level (*p* < 0.001), platelet count (*p* = 0.049), prothrombin time (*p* = 0.004), and hemoglobin A1c level (*p* = 0.013) were significantly correlated with increased index hospital charges.

**TABLE 2:** Demographic and preoperative clinical characteristics and index admission hospital charges for Whipple's PD.

	Patient count *n* (%)	Median index admission total charges (dollar equivalents)	*p*‐value
Age categories			0.277
< 70 years	219 (58%)	0.98	
>/=70 years	156 (42%)	1.02	
Sex			0.532
Male	200 (53%)	0.95	
Female	174 (47%)	1.03	
BMI categories			0.557
</=30	258 (69%)	1.00	
> 30	115 (31%)	0.99	
ASA categories			0.127
1–3	304 (81%)	0.99	
4–5	71 (19%)	1.00	
Smoking history			0.912
Yes	167 (45%)	1.00	
No	202 (55%)	0.99	
Diabetes			0.534
Yes	127 (34%)	1.00	
No	247 (66%)	0.99	
Hypertension			0.984
Yes	208 (56%)	0.98	
No	165 (44%)	1.00	
WBC pre‐op			0.041^a^
< 12000	355 (95%)	0.98	
>/=12000	19 (5%)	1.46	
Hemoglobin pre‐op			< 0.001^a^
>/=13	131 (35%)	0.90	
< 13	244 (65%)	1.09	
Albumin pre‐op			< 0.001^a^
>/=3	344 (92%)	0.98	
< 3	31 (8%)	1.50	
Platelets pre‐op			0.049^a^
>/=150	352 (94%)	0.98	
< 150	23 (6%)	1.21	
PT pre‐op			0.032^a^
</=13.5	181 (49%)	0.98	
> 13.5	187 (51%)	1.00	
Hemoglobin A1c pre‐op			0.020^a^
</=6.5	215 (75%)	0.87	
> 6.5	73 (25%)	1.00	
Pancreatic duct diameter (mm)			0.636
</=2	61 (45.5%)	0.98	
> 2	73 (54.5%)	0.93	
Pancreatic texture			0.464
Soft	58 (43.6%)	1.03	
Firm	75 (56.4%)	0.87	

Abbreviations: ASA, American Society of Anesthesiologists; BMI, body mass index; INR, International Normalized Ratio; PT, Prothrombin Time; WBC, white blood cell.

^a^ Indicates statistical significance (*p* < 0.05).

Notably, albumin, a modifiable laboratory value, showed a significant difference in charges: patients with an albumin level below 3 (*n* = 31) had a median index hospital charge of 0.98 DEs, compared to 1.5 DEs in those with an albumin level of 3 or higher (*n* = 344). Similarly, hemoglobin A1c, another modifiable parameter, also showed significant differences in charges: patients with values in the diabetic range (A1c > 6.5, *n* = 73) had a median charge of 1.0 DEs, compared to 0.87 DEs in those with A1c ≤ 6.5 (*n* = 215). Interestingly, there was no significant difference in charges between patients with and without diabetes (*p* = 0.534): patients with diabetes had a median DE of 1.00, versus 0.99 in patients without a history of diabetes.

Additionally, no significant charge differences were found in patients with advanced age (*p* = 0.277), male sex (*p* = 0.532), elevated BMI (*p* = 0.557), high ASA status (*p* = 0.127), and those with a history of smoking (*p* = 0.912) or hypertension (*p* = 0.984), all of which are predictors of postoperative complications. All demographic and preoperative characteristics, along with their impacts on the total hospital charges, are summarized in Table 2.

### 3.3 Cumulative Preoperative Indicators and Financial Outcomes

**FIGURE 1:**
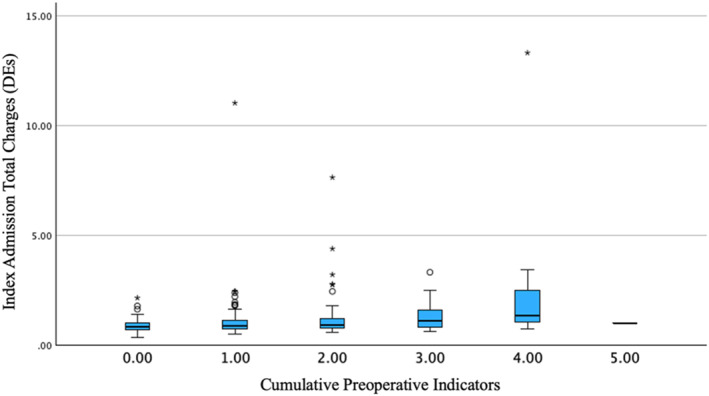
Index admission charges by cumulative preoperative indicators.

Kruskal–Wallis test demonstrated a statistically significant difference in the total index admission hospital charges based on the cumulative number of preoperative indicators (*p* = 0.001), as shown in Figure 1. Among the six identified preoperative indicators of increased hospital charges of index admission, 49 patients had no indicators, and 326 patients had at least one indicator, with no patient having more than five indicators. The median number of preoperative indicators per patient was five. Patients with zero indicators had a median index admission total charge of 0.82 DEs, while those with 5 had a median of 1 DE. The cumulative preoperative indicator distribution and associated hospital charges of patients are summarized in Table 3.

**TABLE 3:** Distribution of patients and median total hospital charges for index admission stratified by cumulative number of preoperative indicators.

Cumulative preoperative indicators	Patient count *n* (%)	Median index admission total charges (dollar equivalents)	*p*‐value
0	55 (19.4%)	0.82	0.001^a^
1	95 (33.5%)	0.88	
2	85 (29.9%)	0.91	
3	37 (13%)	1.11	
4	11 (3.9%)	1.32	
5	1 (0.4%)	1.00	

^a^ Indicates statistical significance (*p* < 0.05), Kruskal–Wallis test.

Notably, subsequent pairwise comparisons revealed significant differences in hospital charges between patients with 0 and 5 preoperative indicators (*p* = 0.046) and between those with 0 and 6 preoperative indicators (*p* = 0.013). No other pairs reached statistical significance. Significance values were adjusted using the Bonferroni correction for multiple tests.

Furthermore, the low‐risk group, classified as those with fewer than three preoperative indicators, had a median total index admission charge of 0.84 DEs, while the high‐risk group, with three or more preoperative indicators, had a median of 0.98 DEs. Mann–Whitney *U* test confirmed a strong association between the risk category and hospital charges (*p* < 0.001), indicating that the high‐risk group, which had a greater number of preoperative indicators, was significantly associated with increased financial burden (Table 4).

**TABLE 4:** Distribution of patients and median total hospital charges for index admission stratified by cumulative preoperative indicators.

Risk categories	Patient count *n* (%)	Median index admission total charges (dollar equivalents)	*p*‐value
Low risk (< 3 preoperative indicators)	235 (82.7%)	0.88	< 0.001^a^
High risk (≥ 3 preoperative indicators)	49 (17.3%)	1.16	

^a^ Indicates statistical significance (*p* < 0.05), Mann–Whitney *U* test.

### 3.4 Secondary Postoperative Outcomes

Among the 375 patients who underwent Whipple's PD during the 5‐year study period, pathologic diagnoses included pancreatic adenocarcinoma in 182 patients (48.5%), pancreatitis in 14 (3.7%), neuroendocrine tumors in 25 (6.7%), benign cystic lesions in 47 (12.5%), cholangiocarcinoma in 29 (7.7%), duodenal or ampullary malignancies in 40 (10.7%), and other pathologies in 36 patients (9.6%). Clinically relevant postoperative pancreatic fistula (CR‐POPF) occurred in 37 patients (9.9%), and 55 patients (14.7%) experienced major complications classified as Clavien–Dindo grade III or IV. The median postoperative length of stay was 8 days (IQR 6–13), and 38 patients (10.1%) required reoperation. The 30‐day mortality rate was 1.3% (*n* = 5), and the 90‐day mortality rate was 1.6% (*n* = 6).

## 4 Discussion

The findings of this retrospective review revealed several key preoperative factors that significantly impact hospital charges of patients undergoing Whipple's PD. These identified factors included preoperative labs: preoperative white blood cell count, hemoglobin, platelet count, and prothrombin time, as well as modifiable values, such as serum albumin and hemoglobin A1c, which were all independently associated with increased hospital charges. These results indicate that baseline physiological status is predictive of not only clinical outcomes but also financial burden in complex surgical care.

The secondary postoperative outcomes in this cohort compare favorably with contemporary benchmarks from high‐volume centers. Major complications (Clavien–Dindo grade III–IV) occurred in 14.7% of the study cohort, CR‐POPF in 9.9%, reoperation in 10.1%, and 30‐day mortality in 1.3%, all of which fall within or under the reported ranges: major complications 13.5%–23%, CR‐POPF 15%–20%, reoperation 6%–12%, 30‐day mortality 1.5%–3.5% [15, 16, 17]. The median LOS of 8 was also within the reported range of 7–9 days, consistent with expected recovery for complex pancreatic surgery [15]. These findings highlight the inherent complexity of PD and provide context for variation in hospital resource utilization. Although postoperative complications were not included as predictors in the primary analysis, their established association with higher costs supports the validity of this study's economic findings.

Importantly, the analysis demonstrated that patients with more of the identified preoperative indicators incurred higher hospital charges, suggesting that the cumulative burden of these preoperative indicators translates into greater postoperative complexity, resulting in increased financial strain. This trend persisted when patients were further stratified into discrete risk categories, suggesting a potential role for these markers in developing predictive cost models.

The results align with previous studies showing associations between comorbidities, complications, and increased hospital charges following major surgeries. Aoki et al. identified several predictors of post‐PD complications, including pancreatic fistula, such as abnormal preoperative values of white blood cells, platelet count, serum albumin, and PT‐INR [10]. Moreover, multiple studies have demonstrated that surgical complications, which frequently lead to prolonged hospital stays, intensive care, and reinterventions, are key drivers of healthcare costs [2, 3, 18, 19, 20, 21, 22]. For instance, Enestvedt et al. found that major complications nearly double the cost of PD, with pharmacy charges accounting for most of the additional costs [23]. The findings of this study build upon previous work by demonstrating that preoperative risk profiles can be used to identify patients at risk of higher costs in addition to complications.

Interestingly, several widely accepted risk factors for postoperative complications, such as male sex, age, increased BMI, high ASA, smoking history, pancreatic duct size, and gland texture, did not show a statistically significant association with increased hospital charges in the analysis [23, 24]. This discrepancy may be a result of using hospital charges rather than true cost data, as charges are often standardized or bundled into fixed packages, masking cost variation from complications. It is also possible that institutional practices, such as standardized perioperative pathways and protocols, mitigate the added costs of complications. Nonetheless, our findings suggest that baseline physiologic status reflected by the identified preoperative laboratory markers has a greater financial impact than other known risk factors for complications in this patient population.

It is important to acknowledge that the association of preoperative hemoglobin, platelet count, and PT with increased hospital charges may partly reflect the additional costs of perioperative blood transfusions and related interventions. While this may not represent a novel finding, it underscores the potential financial impact of correctable hematologic abnormalities and highlights an opportunity for preoperative optimization to potentially reduce both complications and costs.

This has significant implications for value‐based care models as identifying high‐risk patients before surgery can facilitate effective resource allocation and reduction in financial variability. Notably, the identified preoperative indicators are standard preoperative labs that are readily available before surgery and can serve as actionable targets for preoperative planning and perioperative optimization. Interventions targeting modifiable parameters, such as nutritional support or protein supplementation for patients with hypoalbuminemia and improved glycemic control in patients with elevated hemoglobin A1c, can be implemented to decrease hospital costs related to surgery. Since diabetes was not found to be a significant predictor, optimizing HbA1c levels before surgery, regardless of diabetes diagnosis, can help reduce hospitalization costs. Hypoalbuminemia, on the other hand, while it may not always be a modifiable factor prior to surgery, especially when driven by malignancy‐related inflammation, serves as a marker for baseline physiologic status and identifies patients who may benefit from intensified perioperative optimization and resource planning.

These insights also address the impact of rising healthcare costs on patients, particularly through financial toxicity. Early identification of individuals at risk of high costs can inform discussions with patients about anticipated financial burdens and facilitate timely financial counseling. By providing patients with a clearer understanding of potential hospital charges based on their preoperative risk profile, this information can empower them to make more informed decisions regarding the timing of surgery, choice of hospital or surgical approach, and discussions about insurance coverage or financial planning. Preoperative cost prediction not only guides clinicians in optimizing care but also equips patients to actively participate in decisions that may affect both their health outcomes and financial well‐being.

Overall, this study is the first of its kind to examine the healthcare cost of PD based on preoperative factors that may be modified to decrease costs. In addition, this dataset is from a unique real‐world experience at an NUTCC, a type of practice where much of the PD volume is performed.

### 4.1 Limitations

This study had several limitations that warrant consideration. First, the retrospective nature of this study, which lacks randomization and limits the ability to account for confounders such as insurance status and type, along with the single‐center setting, limits the generalizability of the findings. In addition, the analysis did not incorporate readmission and reoperation charges, resulting in an underestimation of the true total charges. Re‐admission costs were excluded to focus specifically on the financial impact of preoperative factors during the index admission, as re‐admissions can be influenced by intra‐ and postoperative events in addition to preoperative factors. Lastly, hospital charges may not accurately reflect true healthcare costs, although they can serve as an institutional measure of the economic burden. Further studies incorporating multicenter data and longitudinal costs of surgical care, including subsequent readmissions and outpatient follow‐up, are needed for a more comprehensive understanding of the financial burden of PD.

## 5 Conclusion

Preoperative laboratory tests are significant predictors of index admission hospital charges in patients undergoing Whipple's PD, some of which are modifiable, such as albumin and A1c. An increased number of these preoperative indicators are associated with increased hospital charges, highlighting the importance of effective risk stratification and preoperative optimization. These findings offer opportunities for improved resource allocation, reduced costs, and enhanced value‐based care for patients undergoing complex surgical procedures.

## Author Contributions

**Sri Snehita Reddy Bonthu:** conceptualization, data curation, visualization, writing – original draft. **Sourodip Mukharjee:** conceptualization, formal analysis, writing – original draft. **Joshua Kong:** writing – review and editing. **Juan Malo:** conceptualization, writing – review and editing. **Houssam Osman:** writing – review and editing. **D. Rohan Jeyarajah:** supervision, writing – review and editing.

## Funding

The authors have nothing to report.

## Conflicts of Interest

Dr. Jeyarajah is a consultant for Ethicon, Sirtex, and Angiodynamics.

## Data Availability

The data that support the findings of this study are available from the corresponding author upon reasonable request.
